# Analysis of the Genetic Variation of the *Fruitless* Gene within the *Anopheles gambiae* (*Diptera*: *Culicidae*) Complex Populations in Africa

**DOI:** 10.3390/insects13111048

**Published:** 2022-11-13

**Authors:** Mahamadi Kientega, Nace Kranjc, Nouhoun Traoré, Honorine Kaboré, Dieudonné Diloma Soma, Ioanna Morianou, Moussa Namountougou, Adrien Marie Gaston Belem, Abdoulaye Diabaté

**Affiliations:** 1 Institut de Recherche en Sciences de la Santé (IRSS), Bobo-Dioulasso 01 BP 545, Burkina Faso; 2 Laboratoire de Santé Animale Tropicale, Institut du Développement Rural, Université Nazi Boni, Bobo-Dioulasso 01 BP 1091, Burkina Faso; 3 Department of Life Sciences, Imperial College, London SW7 2AZ, UK

**Keywords:** *Fruitless*, genomics, *An. gambiae* s.l, vector control, Africa

## Abstract

**Simple Summary:**

In this study, we have characterized the genetic variation of the *Fruitless* gene (X: 1283016-1373662) within 18 populations of *An. gambiae* s.l in Africa. The variant density and the nucleotide diversity were low in the exonic regions of the *fru* gene, especially the male sex-specific region, the BTB-exons 1 and 2, and the zinc-finger B and C exons. These regions also showed high conservation scores. The allelic frequencies of the non-synonymous SNPs were low (freq < 0.26), except for two SNPs identified at high frequencies (freq > 0.8) in the zinc-finger A and B protein domains. These results showed a low genetic variation overall in the exonic regions of the *fru* gene, especially the male sex-specific exon and the BTB-exon 1. These findings are crucial for the development of a gene drive construct targeting the *fru* gene that can rapidly spread without encountering resistance in wild populations.

**Abstract:**

Targeting genes involved in sexual determinism, for vector or pest control purposes, requires a better understanding of their polymorphism in natural populations in order to ensure a rapid spread of the construct. By using genomic data from *An. gambiae* s.l., we analyzed the genetic variation and the conservation score of the *fru* gene in 18 natural populations across Africa. A total of 34,339 SNPs were identified, including 3.11% non-synonymous segregating sites. Overall, the nucleotide diversity was low, and the Tajima’s D neutrality test was negative, indicating an excess of low frequency SNPs in the *fru* gene. The allelic frequencies of the non-synonymous SNPs were low (freq < 0.26), except for two SNPs identified at high frequencies (freq > 0.8) in the zinc-finger A and B protein domains. The conservation score was variable throughout the *fru* gene, with maximum values in the exonic regions compared to the intronic regions. These results showed a low genetic variation overall in the exonic regions, especially the male sex-specific exon and the BTB-exon 1 of the *fru* gene. These findings will facilitate the development of an effective gene drive construct targeting the *fru* gene that can rapidly spread without encountering resistance in wild populations.

## 1. Introduction

In the animal kingdom, the male and female sexes show different morphological and behavioral characteristics. These characteristics are governed by genetics elements that are expressed through various signals to determine the sex of the individual [1]. In insects, sexual dimorphism commonly starts by the primary activation of a central gene that induces molecular cascade mechanisms controlling alternative splicing of the *Doublesex* (*dsx*) and *Fruitless* (*fru*) genes [2,3,4]. Therefore, these two genes (*dsx* and *fru*) are considered as the endpoint in the sex-determination mechanism in insects. Although all the molecular processes involved in the sexual differentiation are less understood, the available data suggest the implication of the *Yob1* gene, a maleness gene located on the *An. gambiae* Y chromosome, as the central gene trigging the male sexual differentiation of the *Anopheles* species [5,6].

In *Drosophila melanogaster* and *Aedes aegypti*, the *fru* gene is expressed in the central nervous system, and it takes part in the construction of a neuronal network to direct the male courtship behavior [2]. The courtship behavior, as complex as it is, regroups a set of behavioral interactions in which the male engages in a succession of actions including orientation and the pursuit of females until they copulate. Studies have also shown the role of the *fru* gene in the construction of a set of abdominal muscles, including the muscle of Lawrence (MOL) that is necessary for copulation [7,8]. In *D. melanogaster*, *fru* mutants show abnormal mating behavior due to the inability to distinguish the partner, a copulation failure, and a weakened abdominal muscle. These mutant males also exhibit a little wing extension, and no song pulse signal was generated when flapping their wings. The ectopic expression of FRU^M^ in *Drosophila* females induced a masculinization of these females with MOL formation. The examination of the abdomen of the *An. gambiae* males compared to those of the females has led to the identification of a sexually dimorphic muscle structure in the A5 abdominal segment, similar to the Lawrence muscle [9]. This muscle structure appears thicker, significantly longer, and more built, with more protruding extension sites, than its corresponding in the female’s A5 abdominal segment. Although the direct involvement of the *fru* gene in *Anopheles* male sexual orientation is less known, previous data recorded from closely related species, notably the *Drosophilidae* species and *Ae. Aegypti*, confirm the role of the *fru* gene in male courtship regulation [8,10].

Comparative analysis of the *fru* gene of the three dipteran species, mainly *An. Gambiae, D. melanogaster*, and *Ae. Aegypti*, has revealed a similar sex-splicing pattern and conservation of certain domains and functions between these species. Comparing the male and female mRNA sequences of the *fru* gene provided insights into the sex-specific splicing pattern in *An. gambiae*. During splicing, the female-specific isoform incorporates an early stop codon, leading to the production of nonfunctional protein. Conversely in males, the sex-splicing pattern excludes the female-specific region, which is producing a transcript that is normally translated into functional protein. This sex-splicing pattern, previously well known in Drosophila species, is shown to be the fundamental element switching the sexual orientation in insect species [8,9].

*An. gambiae* s.l remains one of the main malaria vectors in Africa [11]. It is a complex of nine morphologically undistinguishable species, among which three species (*An. Gambiae s.s., An. coluzzii*, and *An. arabiensis*) well distributed from western to eastern Africa are responsible for almost all the malaria cases in sub-Saharan Africa [11,12]. Current malaria control strategies essentially rely on the use of antimalarial drugs (artemisinin-based combination therapies) against parasites and insecticides (mainly pyrethroids and carbamates) to target vector populations. The intensive use of these tools has contributed significantly to the reduction in malaria incidence, morbidity, and mortality over the past decade [13]. In recent years, due to the rapid rise and generalization of the technical and entomological issues in malaria control, the current control tools seem to have become ineffective and obsolete for reaching malaria elimination [14]. The emergence and rapid spread of insecticides’ resistance, as well as changes in biting and resting behavior and the diversity of the vectoral system in Africa, raise concerns about a dramatic reduction in long-lasting insecticidal nests (LLINs) and the effectiveness of indoor residual sprayings (IRS) [15,16,17,18]. To address these challenges, innovative malaria control tools are being developed to strengthen the current tools and accelerate malaria elimination. For example, genetic control strategies aim to reduce the reproductive and/or vectorial capacity of vectors by distorting the sex ratio, reducing females’ fecundity, or making females unable to transmit pathogens [19]. One of the most promising approaches uses gene drive technologies to reduce the reproductive potential of the mosquito by disrupting the genes essential for female reproduction [19,20,21]. Recently, a gene drive was used to disrupt the female-specific intron-4–exon-5 boundary of the *dsx* gene in the African malaria vector *An. gambiae*. *Dsx* mutant females showed deep morphological abnormalities and were unable to mate or take a blood meal, which are both essential for female reproduction. Releases of *dsx* mutant gene drive mosquitoes in small and large cages resulted in the rapid spread of the transgene and complete *An. gambiae* population suppression in less than a year [20,22]. Based on the success of the *dsx* gene drive, the development of self-sustaining and self-limiting genetic technologies targeting genes of the sex-determination pathway, to reduce the reproductive capacity and population size of vector species, has been a priority. *Fru* is an attractive candidate, since its disruption in *D. melanogaster* and *Ae. aegypti* was shown to alter courtship behavior and reduce successful matings [7,10,23]. For this purpose, it is crucial to target a genomic region showing minimal variability, to ensure the rapid spread of the genetic construct in the field, without the emergence of resistance [20,24]. To this end, we analyzed the diversity and the abundance of single nucleotide polymorphisms (SNPs) in the *fru* gene within *An. gambiae* complex populations, to identify the most conserved regions that would be suitable as gene drive targets. We also analyzed the dynamics of the non-synonymous variants and the conservation score throughout the gene. These findings are crucial for the development of a gene drive construct targeting the *fru* gene that can rapidly spread without encountering resistance in wild populations.

## 2. Materials and Methods

### 2.1. Genomics Data and Mosquito Collection

The genomic data used in this study are from the Ag1000G phase 3 project and were publicly published in February 2021. These data are from 2784 wild-caught *An. gambiae* s.l mosquito species, especially *An. arabiensis*, *An. gambiae s.s.*, and *An. coluzzii*, collected in 19 malaria-endemic countries in sub-Saharan Africa [25]. More details on the mosquito samples’ collection; the sequencing technology used; the storage and the management of the genomic data, including the SNPs variants’ calling, haplotypes’ phasing, and copy number variants’ identification, including the rights to access these data, have been described on the homepage of MalariaGEN [25,26]. Briefly, the mosquito samples were individually sequenced at high coverage using Illumina technology at the Wellcome Sanger Institute. The genomic data were then analyzed using BWA version 0.7.15 and GATK version 3.7-0 to call high-quality SNPs and identify haplotypes and CNVs. After the analyses, the raw sequences in FASTQ format and the aligned sequences in BAM format were stored in the European Nucleotide Archive (ENA). The SNPs, haplotypes, and CNVs in VCF and zarr formats, including the samples’ metadata, have been stored on Google Cloud and are publicly accessible via the malariagen-data package or are directly downloadable [26].

In our study, the access to the SNPs and the haplotypes data was possible through the malariagen-data package, based on the Python programming language, to facilitate access and analysis of genomic data without downloading them. Thus, this package was used to locate and extract SNPs and haplotypes data called in the genomic region of chromosome X (X: 1283016-1373662), corresponding to the *fru* gene of *An. gambiae* s.l. The SNPs and haplotypes data were extracted as multidimensional arrays. The malariagen-data package also allows for access to the reference data of *An. gambiae*, including the reference genome and the transcripts of all the genes identified in the mosquito.

### 2.2. Data Analysis

The SNPs and haplotypes data were analyzed using Python programming on Jupyter Notebooks [27]. Python packages scikit-allel [28], malariagen-data [29], and others standard data-management packages including dask [30], pandas [31], numpy [32], matplotlib [33], and seaborn [34] were then used for the genomic analyses and data manipulation. All the graphs were created by using Python libraries matplotlib, seaborn, and R 4.1.3 [35]. Python 3.8.13 and R 4.1.3 codes for reproducing all analyses in this article are available on GitHub (https://github.com/mkient/fruitless_report) (accessed on 23 October 2022).

#### 2.2.1. Genetic Variation Analysis

The malariagen-data package was used to locate and filter the SNPs and the haplotypes data, called in the genomic region of chromosome X corresponding to the *fru* gene (X: 1283016-1373662). These data were then analyzed using the scikit-allel v1.3.3 package to determine the genetic variation of the gene. Thus, the number of segregating sites, variants’ density, population mutation rate, nucleotide diversity, Tajima’s D neutrality test, and haplotype diversity were calculated over the entire gene and within a 0.5 kb window throughout the *fru* gene. Watterson’s theta estimator (θ) was calculated in the genomic region of the *fru* gene to estimate the mutation rate of the genomic region of the *fru* gene within each *An. gambiae* population. These parameters will provide insights into the genetic diversity of the gene among different anopheline species from different countries. To clarify the evolutionary processes that drive the observed genetic variation in the *fru* region, we performed a chromosome-wide selection scan using the Garud H statistics [36] to detect a signal of positive selection in the chromosome X.

The SNPs’ allele frequencies (the synonymous and non-synonymous SNPs) were calculated within the population using the 5 gene transcripts. Non-synonymous SNPs with maximum allelic frequencies that are greater than 5% in at least one population of *An. gambiae* complex species were defined as major SNPs. The major SNPs were filtered and grouped according to the African regions (West, Central, and East) for the analysis of their heterozygosity dynamics and the pattern of association of these SNPs between each pair of loci. The haplotypes identified at the major non-synonymous SNPs positions were selected for the linkage disequilibrium analysis between each pair of these SNPs. The Lewontin Linkage disequilibrium (D’) [37] was then calculated using the to understand the non-random association between each pair of the major non-synonymous SNPs.

#### 2.2.2. Conservation of the *fru* Gene

The conservation score was obtained for the genomic region of the *fru* gene using the AgamP4 conservation score metric [38]. Conservation score integrates a systematic analysis of genetic variation data from wild populations of *An. gambiae* and synthetic conserved regions of 19 *Anopheles* species, *D. melanogaster*, and two other mosquito species (*Ae. aegypti* and *Cx quinquefasciatus*) that are phylogenetically more distant within the dipteran order. Thus, this program was used to calculate the conservation score of each nucleotide in the *fru* gene and generate a data table containing the conservation score. The data table was then imported into Jupyter notebooks via Pandas to analyze the distribution and the variation of the conservation score alongside and within the specific regions of the gene.

## 3. Results

### 3.1. Genetic Variation within the fru Gene

**Figure A1:**
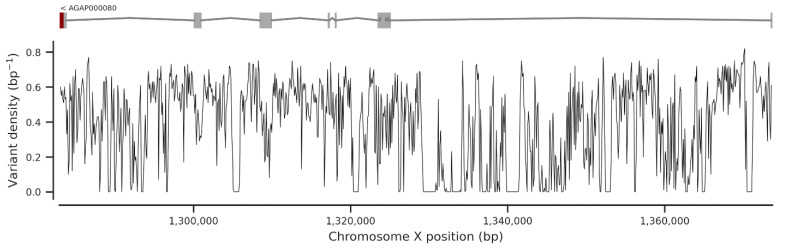
Variant density (bp^−1^) in the genomic region of the *Fruitless* gene; upper figure is the genomic region of the *Fruitless* gene (rectangles correspond to the exonic regions, simple lines are the intronic regions, and dark-red end is the 3 prime UTR); lower figure is the variant density plot.

In total, 34,339 segregating SNPs were identified, including 3.11% (1071 SNPs) non-synonymous segregating sites (750 biallelic non-synonymous variants) and 41.55% (14,268 SNPs) multi-allelic sites (more than two alleles). Many of these SNPs were identified in the intronic region (90.39% (31,042 SNPs)), and only 9.61% (3297 SNPs) were identified in the coding regions of the gene. The lowest SNP variants were identified in the male-specific exons (62 SNPs (0.508 bp^−1^)) and exon 2 (60 SNPs (0.48 bp^−1^)). Globally, the average of the variants’ density was 0.4 bp^−1^, indicating the occurrence of two SNPs in every five bp in the wild populations compared to the reference sequence of the *fru* gene (Figure A1). Compared to previous genome-wide analyses of *An. gambiae* (one SNP for every 1.9 bps) [39], this variant density was slightly lower in the genomic region of the *fru* gene. Table 1 shows the genetic diversity statistics of the *fru* gene, calculated using the SNPs data from each African country that was sampled and *An. gambiae* complex species. From these results, the highest number of SNPs (17,291 SNPs (sample size = 416)) was identified in the *An. gambiae s.s.* species collected in Cameroon.

**Figure A2:**
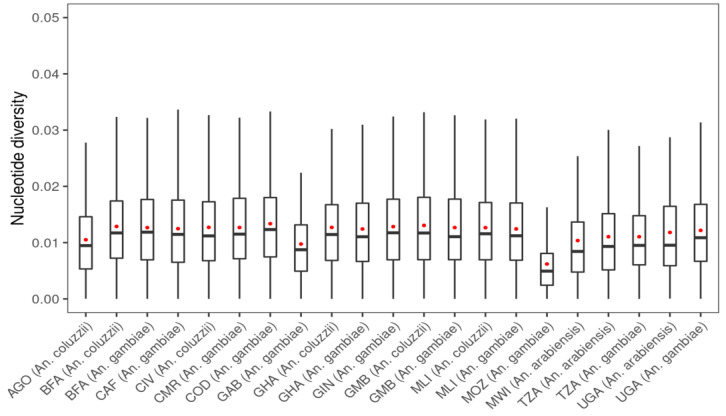
Median of nucleotide diversity in a window of 500 bp across the genomic region of the *Fruitless* gene; red points indicate the mean of the nucleotide diversity; AGO: Angola; BFA: Burkina Faso; CAF: Central African Republic; CIV: Côte d’Ivoire; CMR: Cameroon; COD: Democratic Republic of Congo; GAB: Gabon; GHA: Ghana; GIN: Guinea; GMB: Gambia; MLI: Mali; MOZ: Mozambique; MWI: Malawi; TZA: Tanzania; UGA: Uganda.

**Figure A3:**
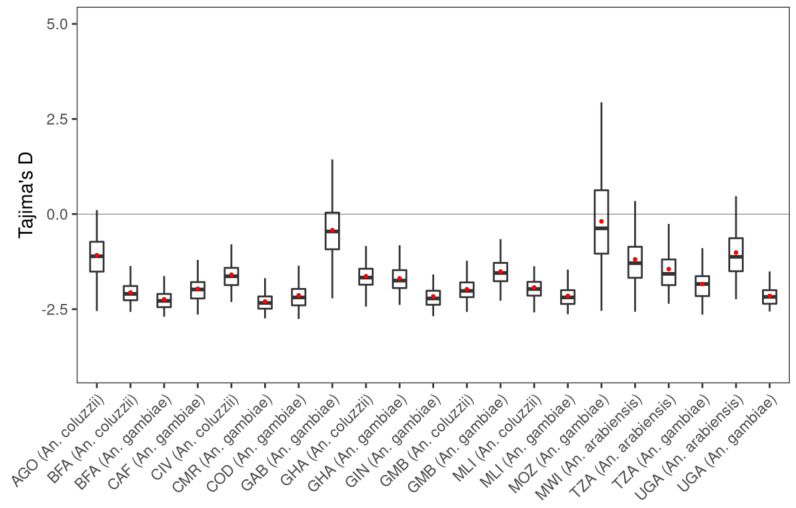
Median of Tajima’s D in a window of 500 bp across the genomic region of the *Fruitless* gene; red points indicate the mean of the Tajima’s D. Horizontal line indicates the null value of the Tajima’s D test. AGO: Angola; BFA: Burkina Faso; CAF: Central African Republic; CIV: Côte d’Ivoire; CMR: Cameroon; COD: Democratic Republic of Congo; GAB: Gabon; GHA: Ghana; GIN: Guinea; GMB: Gambia; MLI: Mali; MOZ: Mozambique; MWI: Malawi; TZA: Tanzania; UGA: Uganda.

The nucleotide diversity is a molecular genomic notion that estimates the level of polymorphism of a genomic region within a population. It measures the number of nucleotide differences per site between two randomly selected DNA sequences in the same population [40]. In our study, the overall nucleotide diversity was 0.0036 in the genomic region of the *fru* gene. Nucleotide diversity, calculated within a non-overlapping 0.5 kb window along the entire *fru* gene, was low in all populations, and the median values ranged from 0.0049 to 0.0157 (Table 1, Figure A2). The overall Tajima’s D test was negative (−2.52) within the genomic region of the *fru* gene (median values of Tajima’s D calculated in a non-overlapping window of 0.5 kb varied from −2.337 to 0.180) (Table 1, Figure A3), indicating an excess of low frequency variants [41]. This low nucleotide diversity and the negative sign of Tajima’s D could be due to an excess of rare variants within the genomic region of the *fru* gene in the *An. gambiae* complex populations. The excess of rare variants in a population may be caused by positive selection within the gene or rapid demographic changes causing the expansion of *An. gambiae* complex populations.

**Figure A4:**
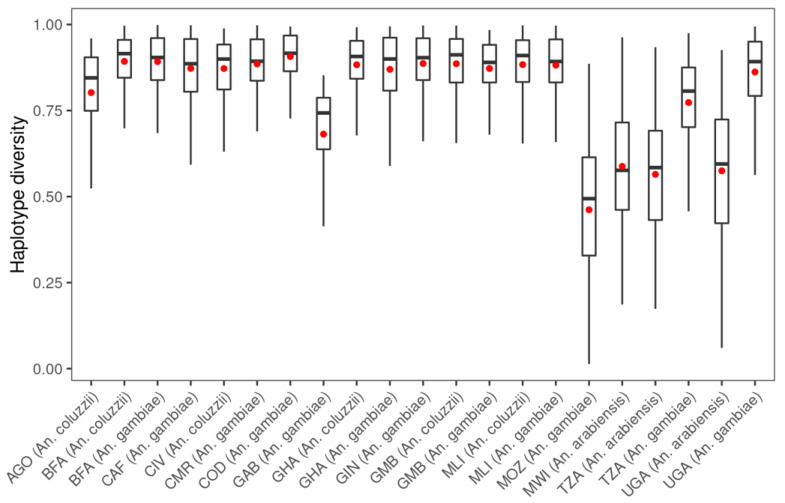
Haplotype diversity in a window of 500 bp across the genomic region of the *Fruitless* gene; red points indicate the mean of the haplotype diversity. AGO: Angola; BFA: Burkina Faso; CAF: Central African Republic; CIV: Côte d’Ivoire; CMR: Cameroon; COD: Democratic Republic of Congo; GAB: Gabon; GHA: Ghana; GIN: Guinea; GMB: Gambia; MLI: Mali; MOZ: Mozambique; MWI: Malawi; TZA: Tanzania; UGA: Uganda.

**Figure A5:**
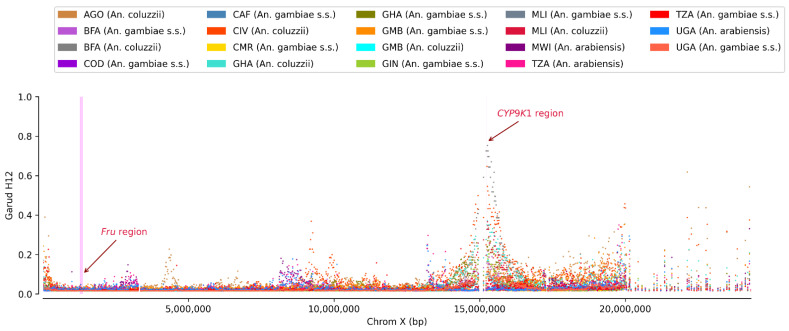
H12 statistic in chromosome X (window size = 4 kb) of *An. gambiae* s.l population. High values of H12 in a given genomic region indicates a signal of positive selection in this genomic region within the population [36]. Magenta bands indicate the *fru* region (X: 1283016-1373662) and the *Cyp9k1* region (X:15240572-15242864). The H12 values were low in the *fru* region, but higher in the *Cyp9k1* region shown to be involved in the pyrethroid resistance in *An. gambiae* s.l populations [42]. AGO: Angola; BFA: Burkina Faso; CAF: Central African Republic; CIV: Côte d’Ivoire; CMR: Cameroon; COD: Democratic Republic of Congo; GHA: Ghana; GIN: Guinea; GMB: Gambia; MLI: Mali; MWI: Malawi; TZA: Tanzania; UGA: Uganda.

To elucidate the evolutionary processes (either a selective sweep or demographic changes) that drive the observed genetic variation, we performed a selection scan at the chromosome X level using the Garud H12 statistics in the *An. gambiae* s.l populations from different regions of Africa. The Garud H12 (<0.05) was low in the *fru* genomic region (Figure A4 and Figure A5). However, compared to the *Cyp9k1* (X:15240572-15242864), a cytochrome P450 gene shown to be involved in pyrethroid resistance [42], the H12 (min = 0.026, median = 0.215, max = 0.782) values were high, suggesting a positive selection in this gene. These results suggest that the excess of rare variants in the population may be caused by rapid demographic changes of *An. gambiae* complex populations.

### 3.2. Non-Synonymous SNPs’ Variation

**Figure 1:**
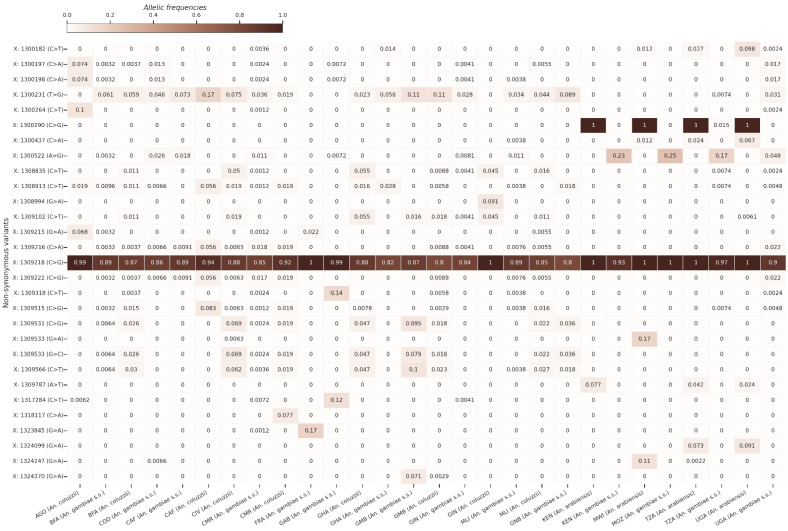
Heat map showing the allelic frequencies of the non-synonymous mutations with maximum allelic frequencies that are > 5% in at least one population. The vertical axis of the heat map shows the non-synonymous variant positions in the X chromosome, and the horizontal axis shows the populations of *An. gambiae* s.l. The gradient color bar shows the distribution of the allelic frequencies. AGO: Angola; BFA: Burkina Faso; CAF: Central African Republic; CIV: Côte d’Ivoire; CMR: Cameroon; COD: Democratic Republic of Congo; FRA: Mayotte; GAB: Gabon; GHA: Ghana; GIN: Guinea; GMB: Gambia; GNB: Guinea-Bissau; MLI: Mali; MOZ: Mozambique; MWI: Malawi; TZA: Tanzania; UGA: Uganda.

Overall, 1071 non-synonymous SNPs were identified, with allelic frequencies ranging from 0 to 1 within the three species of the *An. gambiae* complex (Tables S1 and S2). Figure 1 shows the allelic frequencies of the non-synonymous SNPs with maximum allelic frequencies that are greater than 5% in at least one population. Overall, the allelic frequencies of the non-synonymous SNPs were low, except for two SNPs at positions X:1309218 (C>G) and X:1300290 (C>G), identified at high frequencies in the zinc-finger A and zinc-finger B protein domains, respectively. The SNP at position X:1309218 (C>G) was identified in all the *An. gambiae* complex populations at high allelic frequencies greater than 0.8. However, the SNP at the position X:1300290 (C>G) mutation was only identified in *An. arabiensis* (freq = 1) populations from East Africa. This mutation was also found at low frequency (~0.015) in the *An. gambiae s.s.* population from Tanzania. However, no *An. gambiae* complex population from West or Central Africa has shown this mutation. Its absence in *An. arabiensis* populations from West and Central Africa could be due to the sample size, which is low (Table 1).

**Figure 2:**
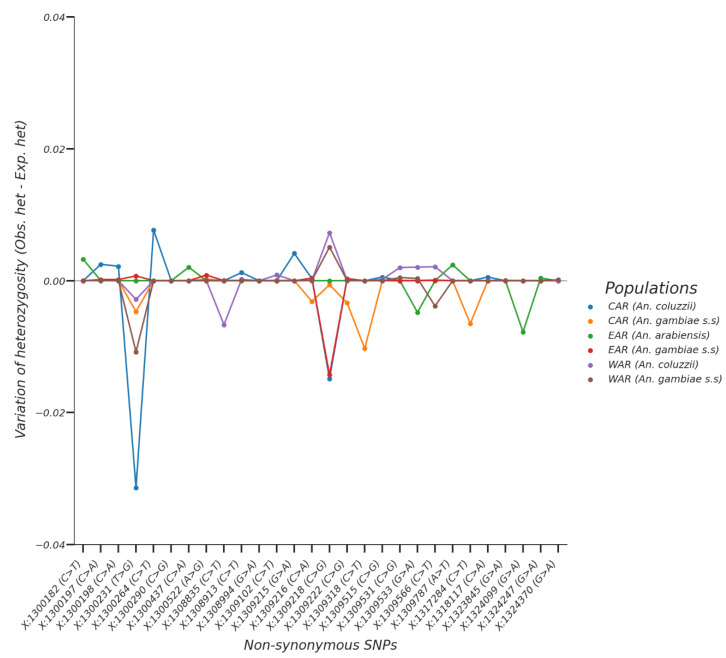
Variation of the heterozygosity within the non-synonymous SNPs with maximum allelic frequencies that are superior to 5% in at least one population of *An. gambiae* complex from West, Central, and East Africa. The vertical axis shows the difference between the observed and the expected heterozygosity; the horizontal axis shows the non-synonymous SNPs positions. Positive values (Obs. het > Exp. het) mean an excess of heterozygotes at this position; negative values (Obs. het < Exp. het) mean a deficit of heterozygotes at this position; null values (Obs. het = Exp. het) mean no deviation from the Hardy–Weinberg equilibrium at this position. Obs. het.: observed heterozygosity, Exp. het.: expected heterozygosity, CAR: Central African Region, EAR: Eastern African Region, WAR: Western African Region.

Analysis of the major SNPs’ (allelic frequencies > 5%) variation has shown variable heterozygosity rates within these SNPs between the *An. gambiae* population. Heterozygosity rates were low and ranged from −0.0313 to 0.0076 (Figure 2), indicating a deviation from the Hardy–Weinberg law at some loci. Indeed, the SNP at position X:1300290 (C>G) showed a very low heterozygosity, indicating that it is fully fixed in the *An. arabiensis* population of East Africa (allelic freq = 1) and is weakly found in the two other *An. gambiae* complex populations. On the other hand, the SNP at position X:1309218 (C>G) has also shown variable heterozygosity, with an excess of heterozygotes in the West African populations (*An. gambiae* and *An. coluzzii*) and a deficit in the Central and East African *An. gambiae* populations. Due to the fixation of the SNP at position X:1309218 (C>G) in the East African *An. arabiensis*, no deviation from the Hardy–Weinberg law was observed at this position. These results are in conformity with the distribution of the allele frequencies that showed the fixation of some non-synonymous SNPs in the populations and a deviation from the Hardy–Weinberg law at some loci.

**Figure 3:**
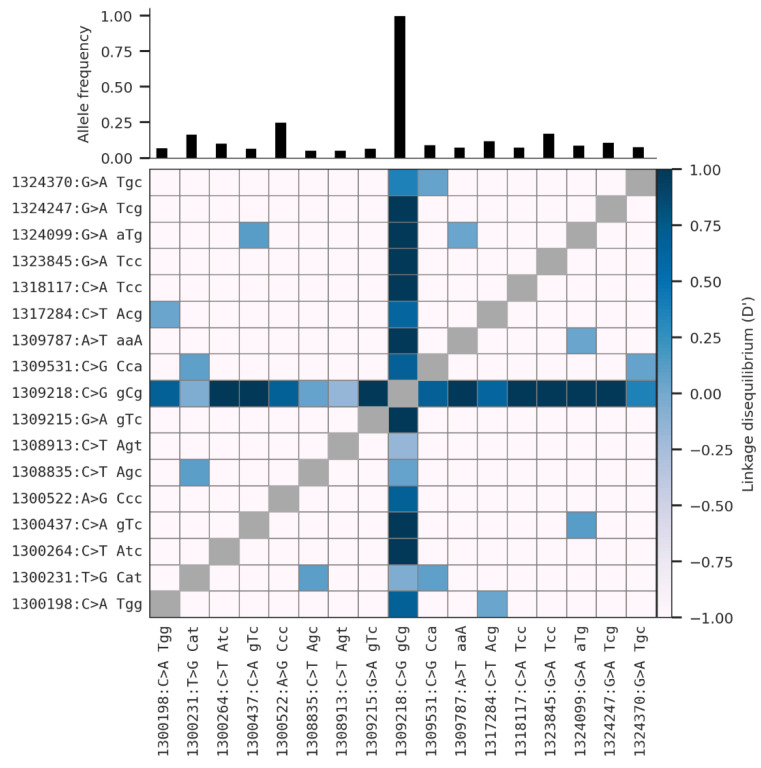
Linkage disequilibrium between the non-synonymous SNPs with maximum allelic frequencies that are greater than 5% in at least one population; upper figure shows allelic frequencies; lower figure shows linkage disequilibrium value (−1 indicates no LD, and +1 indicates perfect LD).

Figure 3 shows the linkage disequilibrium between the major non-synonymous SNPs (max freq > 0.05) and the corresponding allelic frequencies of each SNPs in the whole population. As linkage disequilibrium is the non-random association of alleles at different loci, the mutation at position X:1309218 was strongly associated with the other non-synonymous mutations, exhibiting a perfect linkage disequilibrium with them. This pattern of linkage disequilibrium between the SNP at position X:1309218 and the other SNPs is correlated with the high allelic frequencies in the populations. Overall, genetic variation analysis of the *fru* gene showed a low nucleotide diversity, a negative Tajima’s D, and a strong linkage disequilibrium, as well as a fixation of a SNP at position X:1309218 (C>G) within all the population.

### 3.3. Conservation Score of the fru Gene

**Figure 4:**
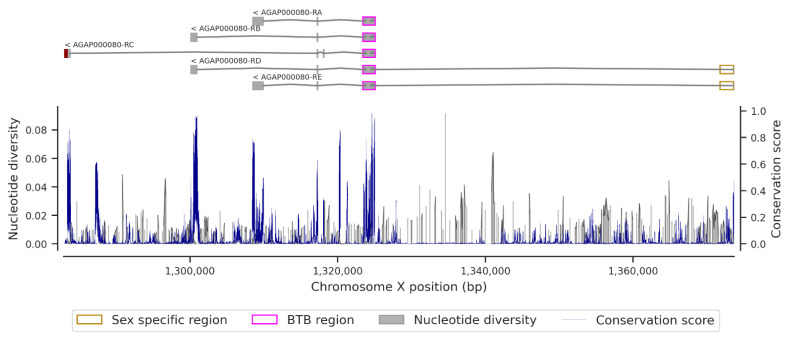
Conservation score and nucleotide diversity in a window of 12 bp within the sex-specific region of the *Fruitless* gene. The upper figure shows the five transcripts of the *Fruitless* gene (rectangles correspond to the exonic regions, simple lines are the intronic regions, and dark-red end is the 3 prime UTR within the AGP000080-RC transcript); lower figure shows the conservation score (blue line) and the nucleotide diversity (dark fill) plot.

**Figure A6:**
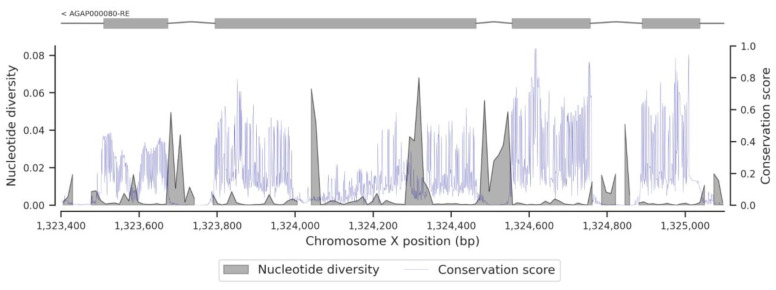
Conservation score and nucleotide diversity in a window of 12 bp within the BTB and connector region of the Fruitless gene; upper figure shows the BTB and connector region (rectangles correspond to the exonic regions, and the simple lines are the intronic regions); lower figure shows the conservation score (blue line) and the nucleotide diversity (dark fill) plot.

**Figure A7:**
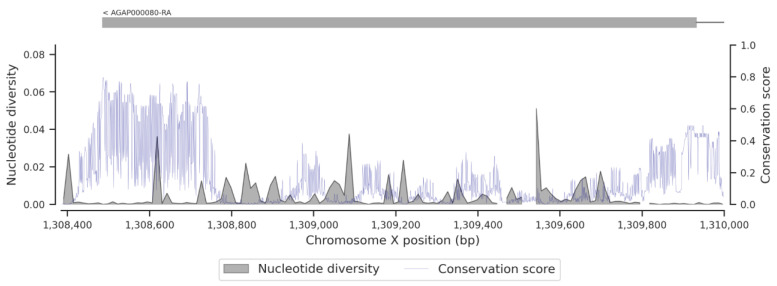
Conservation score and nucleotide diversity in a window of 12 bp within the zinc-finger A (ZnFA) region of the Fruitless gene; upper figure shows the ZnFA region (the rectangle corresponds to the exonic region, and the simple line is the intronic region); lower figure shows the conservation score (blue line) and the nucleotide diversity (dark fill) plot.

**Figure A8:**
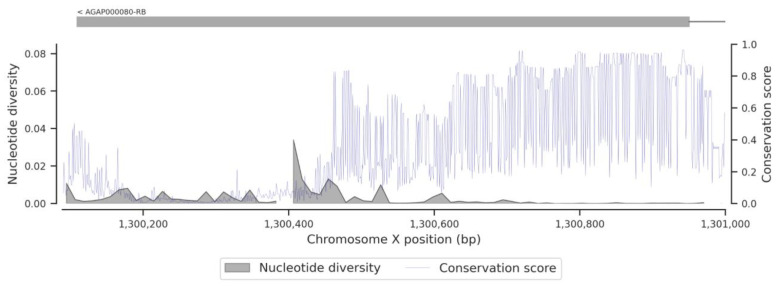
Conservation score and nucleotide diversity in a window of 12 bp within the zinc-finger B (ZnFB) region of the Fruitless gene; upper figure shows the ZnFB region (the rectangle corresponds to the exonic region, and the simple line is the intronic region); lower figure shows the conservation score (blue line) and the nucleotide diversity (dark fill) plot.

**Figure A9:**
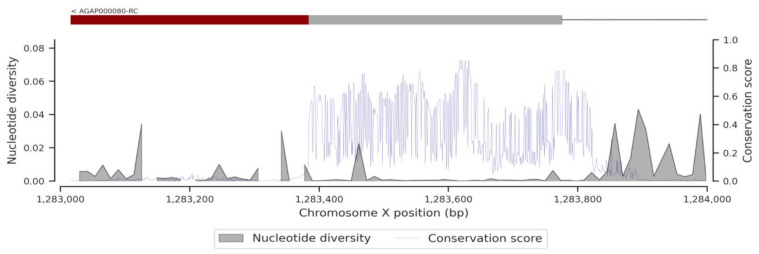
Conservation score and nucleotide diversity in a window of 12 bp within the zinc-finger C (ZnFC) region of the Fruitless gene; upper figure shows the ZnFC region (the rectangle corresponds to the exonic region, the dark red end is the 3 prime UTR region, and the simple line is the intronic region); lower figure shows the conservation score (blue line) and the nucleotide diversity (dark fill) plot.

**Figure A10:**
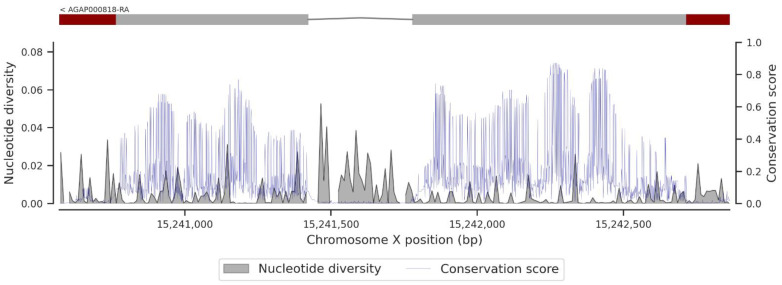
Conservation score and nucleotide diversity in a window of 12 bp within the *Cyp9k1* (X:15240572-15242864), a gene shown to be under selection pressure [36]; upper figure shows the genomic region of the *Cyp9k1* (the rectangle corresponds to the exonic region, the dark red ends are the 3′ and 5′ UTR regions, and the simple line is the intronic region); lower figure shows the conservation score (blue line) and the nucleotide diversity (dark fill) plot. The conservation score remains high in the exonic regions compared to the intronic ones, as it is found in the *fru* gene.

Figure 4 shows the conservation score or the evolution rate of the *fru* gene (Table S3) and the nucleotide diversity within the *fru* gene. In fact, the analyses have shown a high variable of conservation score throughout the *fru* gene, with maximum values in the exonic regions compared to the intronic regions. The median conservation score was 0.00403. High conservation scores (Cs min = 0.00076; Cs median = 0.148; Cs max = 0.983) were recorded in the exonic regions, and these data correlated with the low nucleotide diversity in these genomic regions (Figure 4, Figure A6, Figure A7, Figure A8 and Figure A9). The same pattern of conservation was observed in the exonic regions of *Cyp9k1* (Cs min = 0.00019; Cs median = 0.101; Cs max = 0.871), a cytochrome P450 gene under strong selective pressure, shown to be involved in pyrethroid resistance [42] (Figure A10).

**Figure 5:**
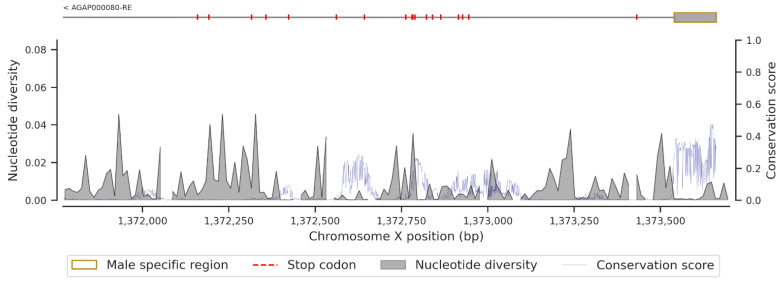
Conservation score and nucleotide diversity in a window of 12 bp within the sex-specific region of the *Fruitless* gene. The upper figure is the sex-specific region of the *Fruitless* gene (rectangle corresponds to the male-specific region, rectangle and simple line correspond to the female-specific region, and red dashes are the probable stop codon within the female-specific region); lower figure is the conservation score (blue line) and the nucleotide diversity (dark fill) plot.

Furthermore, the female-specific region of the *fru* gene spans the region X:1371771-1373662 and contains stop codons (Figure 5) that stop the mRNA translation during protein synthesis from rendering the *fru* gene non-functional in females. These stop codons are responsible for the early termination of mRNA translation, which makes the female FRU isoform non-functional. The female-specific region includes a short exon (~122 bp) at its beginning and spanning region X:1373540-1373662 (Figure 5) on the X chromosome corresponding to the male-specific region. This exon is translated into protein and makes the gene functional in males. In the male-specific region, the conservation score was relatively high (Cs min = 0.0316; Cs median = 0.321; Cs max = 0.475) and, conversely, a low nucleotide diversity was noted in this region. These results showed a high conservation score of the genomic region common to both male and female transcripts compared to the non-common region, which exhibited a relatively low conservation score (Cs min = 0; Cs median = 0.006; Cs max = 0.286) and high nucleotide diversity at some locations. The BTB (broad complex, tramtrack, and bric-à-brac) domain is responsible for protein–protein interaction participating in a wide range of cellular functions in the organism. This domain, primarily identified in the fruit fly [43], is also found in the *An. gambiae* FRU protein, and its genomic region spans from X:1323509 to X:1325039. It is common in the male and female transcripts and has four exons encoding the BTB domain. The conservation scores (Cs min = 0.004; Cs median = 0.169; Cs max = 0.983) computed in the exons of the BTB region were relatively high compared to those of the intronic regions (Figure A6). Conversely, the nucleotide diversity remains high in the intronic regions and in some windows of exon 3. Seeing the pattern of the conservation score, the coding regions are highly conserved compared to the non-coding regions, as shown by previous studies [8,9].

## 4. Discussion

The genetic control of vectors has instigated a major interest in the study of the genetic mechanisms involved in the sexual determination and differentiation for the control of a pest insect’s population, particularly the disease vectors. Thus, the *fru* and *dsx* genes are potential targets for controlling sexual behavior and the differentiation of malaria vectors, respectively. However, the success of a genome editing construct targeting these genes in *An. gambiae* s.l. requires perfect insights into the genetic polymorphism of the target regions in wild populations, in order to avoid resistance to the spread of the construct. Our study analyzed the distribution and the abundance of SNPs within the *fru* gene in 18 populations of the *An. gambiae* complex in Africa.

The SNPs’ distribution across the *fru* gene was variable, and almost all the SNPs were identified in intronic regions. The average SNPs’ density (two SNPs per five bp) was low compared to those previously found in the whole genome of *An. gambiae* s.l. [44]. Indeed, according to the first published *An. gambiae* s.l. genome data, chromosomes 2 and 3 exhibited high SNPs’ density compared to the X chromosome [45]. In addition, the intronic regions showed a high level of polymorphism compared to the exons, and only 9.60% of SNPs variants including two non-synonymous SNPs with high frequencies, X:1309218 C>G (G669A) and X:1300290 C>G (G651A), were identified within the exons. These two SNPs, although their effect is moderate, may have an impact on the structure of the resulting protein. Both SNPs cause the change of C>G at positions X:1309218 and X:1300290, leading to amino acids’ change from glycine to alanine at positions 651 (G651A) and 669 (G669A), respectively. The SNPs identified in the non-coding regions do not directly affect the protein sequence but may have some effects on the regulation of transcription and the gene activity. Indeed, the spliceosome elements interact with specific sites on the intron and exon terminals to ensure efficient and specific splicing [46,47]. Thus, a high density of SNPs in these regions can lead to the non-recognition of the interaction sites, resulting in a dysfunction of the regulation process of the gene expression.

Our results also showed a signal of population expansion causing an excess of low-frequency SNPs, low nucleotide diversity, and negative Tajima’s D in all populations. Although previous studies have shown a signal of positive selection in insecticide-resistance genes [48,49], the Garud H12 [36] was low in the *fru* region, suggesting that all the genetic variation found in the *fru* region is probably caused by evolutionary processes other than positive selection. The rapid demographic changes can also cause an excess of rare variants in a given genomic region [50], as shown in our study, but additional population genomic studies would be needed to clarify that. Considering the SNP X:1300290 (C>G) (Figure 1), only identified at a high frequency in *An. arabiensis* populations from East Africa, the role of evolutionary processes in the maintenance of certain advantageous SNPs at a high frequency in the populations of the *An. gambiae* complex is very clear. The same SNP was identified at a low frequency in *An. gambiae* s.l populations (freq = 0.015 (sample size = 68)) in Tanzania. The existence of a possible introgression process between *An. gambiae s.s.* and *An. arabiensis*, two subspecies of the *An. gambiae* complex, is the subject of considerable debate in the scientific community. A simulation study has demonstrated the occurrence of a possible gene flow between *An. gambiae s.s* and *An. arabiensis*, which still remains to be confirmed in wild populations [51]. The low frequency of SNP X:1300290 (C>G) in the two other populations could have two possible explanations: either as a purifying selection against this mutation in *An. gambiae s.s.* and *An. coluzzii* or as a possible gene flow between *An. gambiae s.s.* and *An. arabiensis*, as predicted by previous studies [51,52]. However, further studies are still needed to confirm or contradict these hypotheses.

The genomic organization of the *fru* gene has been extensively studied in several *Diptera* species, and the results of these studies have shown a strong conservation of the specific domains of the *fru* gene, notably the BTB and the zinc-finger A, B, and C domains between the *An. gambiae* s.l, *D. melanogaster*, and *Ae. aegypti* species [8,9]. In our study, the conservation score corresponding to the evolution rate of the *fru* gene was variable, and the exonic regions seemed to be more conserved compared to introns. Although the algorithm only analyzes nucleotide sequences, the distribution of the conservation score alongside the *fru* gene follows the same conservation pattern highlighted by previous analyses, with a high level of conservation (>70%) in the coding regions of the BTB and the zinc-finger A, B, and C domains of the FRU protein. However, a comparative analysis of sex-specific protein domains has revealed a low conservation of these regions between the *Drosophila, Ae. aegypti*, and *An. gambiae* species, which could be due to the alternative splicing process that still remains specific in each of these Dipteran species [8]. The conservation score of the male-specific genomic region of the *fru* gene was moderate (>30%), indicating a low level of conservation of this region between *Anopheles* species and other Dipteran (*Culex* sp., *Aedes* sp., and *Drosophila* sp.). These results confirm the previous findings that showed a strong divergence between the genomes of these species [8,9].

In the purpose of vector control, the concept of using gene drive technologies to target and modify a given genomic region in the vector genome in order to disrupt progeny and reduce vector density is recurrent [19]. Although successful modifications have been performed in the *An. gambiae* and *Ae. aegypti* genomes, the development of resistance in the target site could strongly affect the long-term spread of the constructs in nature. Indeed, the spread of a transgene in nature requires a quasi-low polymorphism in the target region [53,54]. Thus, a thorough insight into the polymorphism and the evolution rate of the target regions is essential before any genetic modification action. In our study, the density of variants and nucleotide diversity within the *fru* gene were low in 18 populations of *An. gambiae* s.l in Africa, mainly in the male sex-specific region (Figure 5), the BTB-exons 1 and 2 (Figure A6), and the zinc-finger B (Figure A8) and C (Figure A9) exons. These findings will facilitate the development of an effective gene drive construct targeting the *fru* gene that can rapidly spread without encountering resistance in wild populations.

## Figures and Tables

**Table 1:** Summary of the diversity statistics based on SNPs of the *Fruitless* gene of *Anopheles gambiae* complex. Segregating sites were calculated within the whole gene. Median of π, D, θ, and H calculated in a window of 0.5 kb.

Populations	Number of Mosquitoes	SNPs	ns SNPs	Biallelic ns SNP	π	D	θ	H
AGO (*An. coluzzii*)	81	2280	96	95	0.0095	−1.109	0.014	0.845
BFA (*An. arabiensis*)	3	289	2	2	0.0088	−0.256	0.008	0.600
BFA (*An. coluzzii*)	135	8312	118	114	0.0118	−2.098	0.037	0.915
BFA (*An. gambiae s.s.*)	157	11,035	212	202	0.0119	−2.282	0.046	0.904
CAF (*An. coluzzii*)	18	2372	16	16	0.0108	−1.469	0.018	0.910
CAF (*An. gambiae s.s.*)	55	5303	38	38	0.0115	−1.983	0.029	0.886
CIV (*An. coluzzii*)	80	4440	38	37	0.0112	−1.635	0.023	0.900
CMR (*An. arabiensis*)	2	283	2	2	0.0157	0.180	0.015	0.833
CMR (*An. coluzzii*)	26	259	19	18	0.0104	−1.407	0.017	0.897
CMR (*An. gambiae s.s.*)	416	17,291	430	392	0.0115	−2.337	0.059	0.893
COD (*An. gambiae s.s.*)	76	7955	172	167	0.0123	−2.192	0.039	0.916
FRA (*An. gambiae s.s.*)	23	966	56	56	0.0050	−1.164	0.008	0.618
GAB (*An. gambiae s.s.*)	69	1348	20	19	0.0087	−0.458	0.010	0.743
GHA (*An. coluzzii*)	64	4285	34	34	0.0114	−1.670	0.024	0.907
GHA (*An. gambiae s.s.*)	36	3685	18	18	0.0110	−1.749	0.024	0.900
GIN (*An. coluzzii*)	11	930	5	5	0.0095	−0.258	0.009	0.835
GIN (*An. gambiae s.s.*)	123	9083	118	111	0.0118	−2.218	0.040	0.904
GMB (*An. coluzzii*)	169	8241	92	91	0.0117	−2.019	0.034	0.912
GMB (*An. gambiae s.s.*)	69	3952	37	35	0.0111	−1.549	0.022	0.890
GNB (*An. gambiae s.s.*)	29	3375	32	32	0.0117	−1.696	0.022	0.900
KEN (*An. gambiae s.s.*)	28	1221	44	44	0.0074	−1.001	0.010	0.732
MLI (*An. arabiensis*)	2	188	0	0	0.0090	0.135	0.008	0.667
MLI (*An. coluzzii*)	91	6451	67	67	0.0116	−1.970	0.032	0.910
MLI (*An. gambiae s.s.*)	131	8888	125	121	0.0112	−2.191	0.041	0.893
MOZ (*An. gambiae s.s.*)	74	651	6	6	0.0049	−0.375	0.005	0.494
MWI (*An. arabiensis*)	41	1381	15	15	0.0084	−1.290	0.014	0.576
TZA (*An. arabiensis*)	225	2317	41	41	0.0093	−1.571	0.019	0.584
TZA (*An. gambiae s.s.*)	68	4066	71	70	0.0095	−1.838	0.025	0.807
UGA (*An. arabiensis*)	82	1545	26	26	0.0095	−1.124	0.015	0.595
UGA (*An. gambiae s.s.*)	207	10,083	120	114	0.0109	−2.177	0.042	0.892

π: nucleotide diversity; D: Tajima’s D; θ: Watterson’s theta (population mutation rate); H: haplotype diversity; ns SNPs: non-synonymous single nucleotide polymorphism; AGO: Angola; BFA: Burkina Faso; CAF: Central African Republic; CIV: Côte d’Ivoire; CMR: Cameroon; COD: Democratic Republic of Congo; FRA: Mayotte; GAB: Gabon; GHA: Ghana; GIN: Guinea; GMB: Gambia; GNB: Guinea-Bissau; KEN: Kenya; MLI: Mali; MOZ: Mozambique; MWI: Malawi; TZA: Tanzania; UGA: Uganda.

## Data Availability

Python 3.8.13 and R 4.1.3 codes to reproduce all the analyses in the article are available from GitHub: https://github.com/mkient/fruitless_report (accessed on 23 October 2022). The SNPs and haplotypes data are available on the homepage of MalariaGEN and can be accessed using the malariagen-data package.

## Supplementary Materials

The following supporting information can be downloaded at: https://www.mdpi.com/article/10.3390/insects13111048/s1. Table S1: All variants’ allelic frequencies. pos: chromosome X position, wt_ac: wild type alleles count, alt1_ac: alternative 1 alleles count, alt2_ac: alternative 2 alleles count, alt3_ac: alternative 3 alleles count, wt_af: wild type alleles allelic frequencies, alt1_af: alternative 1 allele allelic frequencies, alt2_af: alternative 2 alleles allelic frequencies, alt3_af: alternative 3 alleles allelic frequencies. Table S2: Nonsynonymous allelic frequencies. pos: chromosome X position, wt_ac: wild type alleles count, alt1_ac: alternative 1 alleles count, alt2_ac: alternative 2 alleles count, alt3_ac: alternative 3 alleles count, wt_af: wild type alleles allelic frequencies, alt1_af: alternative 1 allele allelic frequencies, alt2_af: alternative 2 alleles allelic frequencies, alt3_af: alternative 3 alleles allelic frequencies. Table S3: Conservation score within the Fruitless gene. pos: chromosome X position, Cs: Conservation score.

## References

[1] Sánchez L. (2008). Sex-determining mechanisms in insects. Int. J. Dev. Biol..

[2] Siwicki K.K., Kravitz E.A. (2009). *Fruitless*, *Doublesex* and the genetics of social behavior in *Drosophila melanogaster*. Curr. Opin. Neurobiol..

[3] Salvemini M., Mauro U., Lombardo F., Milano A., Zazzaro V., Arcà B., Polito L.C., Saccone G. (2011). Genomic organization and splicing evolution of the *Doublesex* gene, a *Drosophila* regulator of sexual differentiation, in the dengue and yellow fever mosquito *Aedes aegypti*. BMC Evol. Biol..

[4] Biedler J.K., Tu Z. (2016). Sex determination in mosquitoes. Advances in Insect Physiology.

[5] Krzywinska E., Krzywinski J. (2018). Effects of stable ectopic expression of the primary sex determination gene yob in the mosquito *Anopheles gambiae*. Parasites Vectors.

[6] Krzywinska E., Dennison N.J., Lycett G.J., Krzywinski J. (2016). A maleness gene in the malaria mosquito *Anopheles gambiae*. Science.

[7] Gailey D.A., Taylor B.J., Hall J.C. (1991). Elements of the *Fruitless* locus regulate development of the muscle of lawrence, a male-specific structure in the abdomen of *Drosophila melanogaster* adults. Development.

[8] Salvemini M., D’Amato R., Petrella V., Aceto S., Nimmo D., Neira M., Alphey L., Polito L.C., Saccone G. (2013). The orthologue of the fruitfly sex behaviour gene *Fruitless* in the mosquito *Aedes aegypti*: Evolution of genomic organisation and alternative splicing. PLoS ONE.

[9] Gailey D.A., Billeter J.-C., Liu J.H., Bauzon F., Allendorfer J.B., Goodwin S.F. (2005). Functional conservation of the *Fruitless* male sex-determination gene across 250 myr of insect evolution. Mol. Biol. Evol..

[10] Yamamoto D., Kohatsu S. (2017). What does the *Fruitless* gene tell us about nature vs. nurture in the sex life of *Drosophila*?. Fly.

[11] Carnevale P., Robert V. (2009). Les anophèles: Biologie, transmission du Plasmodium et lutte antivectorielle.

[12] Coetzee M. (2020). Key to the females of afrotropical *Anopheles mosquitoes* (*Diptera: Culicidae*). Malar. J..

[13] Bhatt S., Weiss D.J., Cameron E., Bisanzio D., Mappin B., Dalrymple U., Battle K.E., Moyes C.L., Henry A., Eckhoff P.A. (2015). The effect of malaria control on *Plasmodium falciparum* in Africa between 2000 and 2015. Nature.

[14] Ranson H. (2017). Current and future prospects for preventing malaria transmission via the use of insecticides. Cold Spring Harb. Perspect. Med..

[15] Benelli G., Beier J.C. (2017). Current vector control challenges in the fight against malaria. Acta Trop..

[16] Coleman M., Hemingway J., Gleave K.A., Wiebe A., Gething P.W., Moyes C.L. (2017). Developing global maps of insecticide resistance risk to improve vector control. Malar. J..

[17] Sougoufara S., Doucouré S., Sembéne P.M.B., Harry M., Sokhna C. (2017). Challenges for malaria vector control in sub-saharan africa: Resistance and behavioral adaptations in *Anopheles* populations. J. Vector Borne Dis..

[18] Bamou R., Mbakop L.R., Kopya E., Ndo C., Awono-Ambene P., Tchuinkam T., Rono M.K., Mwangangi J., Antonio-Nkondjio C. (2018). Changes in malaria vector bionomics and transmission patterns in the equatorial forest region of Cameroon between 2000 and 2017. Parasites Vectors.

[19] Adelman Z.N., Tu Z. (2016). Control of mosquito-borne infectious diseases: Sex and gene drive. Trends Parasitol..

[20] Kyrou K., Hammond A.M., Galizi R., Kranjc N., Burt A., Beaghton A.K., Nolan T., Crisanti A. (2018). A CRISPR–Cas9 gene drive targeting *Doublesex* causes complete population suppression in caged *Anopheles gambiae* mosquitoes. Nat. Biotechnol..

[21] Hammond A., Galizi R., Kyrou K., Simoni A., Siniscalchi C., Katsanos D., Gribble M., Baker D., Marois E., Russell S. (2016). A CRISPR-Cas9 gene drive system targeting female reproduction in the malaria mosquito vector *Anopheles gambiae*. Nat. Biotechnol..

[22] Hammond A., Pollegioni P., Persampieri T., North A., Minuz R., Trusso A., Bucci A., Kyrou K., Morianou I., Simoni A. (2021). Gene-drive suppression of mosquito populations in large cages as a bridge between lab and field. Nat. Commun..

[23] Basrur N.S., de Obaldia M.E., Morita T., Herre M., von Heynitz R.K., Tsitohay Y.N., Vosshall L.B. (2020). *Fruitless* mutant male mosquitoes gain attraction to human odor. Elife.

[24] Hammond A., Karlsson X., Morianou I., Kyrou K., Beaghton A., Gribble M., Kranjc N., Galizi R., Burt A., Crisanti A. (2021). Regulating the expression of gene drives is key to increasing their invasive potential and the mitigation of resistance. PLOS Genet..

[25] The Anopheles gambiae 1000 Genomes Consortium Ag1000G Phase 3 SNP Data Release. https://www.malariagen.net/data/ag1000g-phase3-snp.

[26] MalariaGEN Malariagen Vector Data User Guide. https://malariagen.github.io/vector-data/landing-page.html.

[27] Kluyver T., Ragan-Kelley B., Pérez F., Granger B., Bussonnier M., Frederic J., Kelley K., Hamrick J., Grout J., Corlay S. (2016). Jupyter Notebooks—A publishing format for reproducible computational workflows. Positioning and Power in Academic Publishing: Players, Agents and Agendas.

[28] Miles A., Bot P., Murillo R., Ralph P., Harding N., Pisupati R., Rae S., Millar T. Cggh/Scikit-Allel, Version 1.3.3; Zenodo 2021. https://zenodo.org/record/4759368#.Y3BX0-RBw2w.

[29] Miles A. GitHub—Malariagen/Malariagen-Data-Python: A Python Package Providing Functions for Accessing and Analysing MalariaGEN Data. https://github.com/malariagen/malariagen-data-python.

[30] Rocklin M. Dask: Parallel computation with blocked algorithms and task scheduling. Proceedings of the 14th Python in Science Conference.

[31] McKinney W. Data structures for statistical computing in python. Proceedings of the 9th Python in Science Conference.

[32] Harris C.R., Millman K.J., van der Walt S.J., Gommers R., Virtanen P., Cournapeau D., Wieser E., Taylor J., Berg S., Smith N.J. (2020). Array programming with numpy. Nature.

[33] Hunter J.D. (2007). Matplotlib: A 2D graphics environment. Comput. Sci. Eng..

[34] Waskom M., Botvinnik O., O’Kane D., Hobson P., Lukauskas S., Gemperline D.C., Augspurger T., Halchenko Y., Cole J.B., Warmenhoven J. Mwaskom/Seaborn, Version 0.8.1; Zenodo 2017. https://zenodo.org/record/883859#.Y3BVeuRBw2w.

[35] R Core Team (2021). R: The R Project for Statistical Computing.

[36] Garud N.R., Messer P.W., Buzbas E.O., Petrov D.A. (2015). Recent selective sweeps in North American *Drosophila melanogaster* show signatures of soft sweeps. PLoS Genet..

[37] Lewontin R.C. (1964). The interaction of selection and linkage. I. general considerations; heterotic models. Genetics.

[38] Kranjc N., Crisanti A., Nolan T., Bernardini F. (2021). *Anopheles gambiae* genome conservation as a resource for rational gene drive target site selection. Insects.

[39] The *Anopheles gambiae* 1000 Genomes Consortium (2017). Genetic diversity of the African malaria vector *Anopheles gambiae*. Nature.

[40] Nei M., Li W.H. (1979). Mathematical model for studying genetic variation in terms of restriction endonucleases. Proc. Natl. Acad. Sci. USA.

[41] Tajima F. (1989). Statistical method for testing the neutral mutation hypothesis by DNA polymorphism. Genetics.

[42] Vontas J., Grigoraki L., Morgan J., Tsakireli D., Fuseini G., Segura L., de Carvalho J.N., Nguema R., Weetman D., Slotman M.A. (2018). Rapid selection of a pyrethroid metabolic enzyme *Cyp9k1* by operational malaria control activities. Proc. Natl. Acad. Sci. USA.

[43] Zollman S., Godt D., Privé G.G., Couderc J.L., Laski F.A. (1994). The BTB domain, found primarily in zinc finger proteins, defines an evolutionarily conserved family that includes several developmentally regulated genes in *Drosophila*. Proc. Natl. Acad. Sci. USA.

[44] The *Anopheles gambiae* 1000 Genomes Consortium (2020). Genome variation and population structure among 1142 mosquitoes of the African Malaria Vector Species *Anopheles gambiae* and *Anopheles coluzzii*. Genome Res..

[45] Holt R.A., Subramanian G.M., Halpern A., Sutton G.G., Charlab R., Nusskern D.R., Wincker P., Clark A.G., Ribeiro J.C., Wides R. (2002). The genome sequence of the malaria mosquito *Anopheles gambiae*. Science.

[46] de Roos A.D.G. (2005). Origins of introns based on the definition of exon modules and their conserved interfaces. Bioinformatics.

[47] Rogozin I.B., Carmel L., Csuros M., Koonin E. (2012). V Origin and evolution of spliceosomal introns. Biol. Direct.

[48] Grau-Bové X., Tomlinson S., O’Reilly A.O., Harding N.J., Miles A., Kwiatkowski D., Donnelly M.J., Weetman D. (2020). Evolution of the insecticide target *rdl* in African *Anopheles* is driven by interspecific and interkaryotypic introgression. Mol. Biol. Evol..

[49] Clarkson C.S., Miles A., Harding N.J., O’Reilly A.O., Weetman D., Kwiatkowski D., Donnelly M.J. (2021). The genetic architecture of target-site resistance to pyrethroid insecticides in the African malaria vectors *Anopheles gambiae* and *Anopheles coluzzii*. Mol. Ecol..

[50] Sætre G.-P., Ravinet M. (2019). Evolutionary Genetics: Concepts, Analysis, and Practice.

[51] Weetman D., Steen K., Rippon E.J., Mawejje H.D., Donnelly M.J., Wilding C.S. (2014). Contemporary gene flow between wild *An*. *gambiae* s.s. and *An. arabiensis*. Parasites Vectors.

[52] Neafsey D.E., Lawniczak M.K.N., Park D.J., Redmond S.N., Coulibaly M.B., Traoré S.F., Sagnon N., Costantini C., Johnson C., Wiegand R.C. (2010). SNP genotyping defines complex gene-flow boundaries among African malaria vector mosquitoes. Science.

[53] Champer J., Liu J., Oh S.Y., Reeves R., Luthra A., Oakes N., Clark A.G., Messer P.W. (2018). Reducing resistance allele formation in CRISPR gene drive. Proc. Natl. Acad. Sci. USA.

[54] Unckless R.L., Clark A.G., Messer P.W. (2017). Evolution of resistance against CRISPR/Cas9 gene drive. Genetics.

